# Association of C-reactive protein with mortality in Covid-19 patients: a secondary analysis of a cohort study

**DOI:** 10.1038/s41598-023-47680-x

**Published:** 2023-11-21

**Authors:** Fei Li, Mingjun He, Mingchao Zhou, Yuyao Lai, Yongjie Zhu, Ziji Liu, Yulong Wang, Yao Wang

**Affiliations:** 1 Department of Rehabilitation Medicine, Dapeng New District Nan’ao People’s Hospital, No. 6, Renmin Road, Nanao Street, Dapeng New District, Shenzhen, 518121 Guangdong China; 2 Department of Rehabilitation, Futian District, Shenzhen Second People’s Hospital/Health Science Centre, The First Affiliated Hospital, School of Medicine, Shenzhen University, No. 3002, Sungang Road, Shenzhen, 518035 Guangdong China

**Keywords:** Viral infection, Influenza virus

## Abstract

Our study aimed to explore the association between serum C-reactive protein (CRP) and COVID‐19 mortality. This is a retrospective cohort study of all patients admitted to 4 hospitals within the Montefiore Health System between March 1 and April 16, 2020, with SARS-CoV-2 infection. All-cause mortality were collected in 7 May 2020. The mortality risk was estimated using Cox proportional hazards models. Of the 3545 patients with a median age of 63.7 years, 918 (25.9%) died within the time of cohort data collection after admission. When the CRP was < 15.6 mg/L, the mortality rate increased with an adjusted HR of 1.57 (95% CI 1.30–1.91, P < 0.0001) for every 10 mg/L increment in the CRP. When the CRP was ≥ 15.6 mg/L, the mortality rate increased with an adjusted HR of 1.11 (95% CI 0.99–1.24, P = 0.0819) for every 10 mg/L increment in the CRP. For patients with COVID‐19, the association between the CRP and the mortality risk was curve and had a saturation effect. When the CRP was small, the mortality rate increased significantly with the increase of CRP. When CRP > 15.6 mg/L, with the increase of CRP, the mortality rate increases relatively flat.

## Introduction

At the end of 2019, Global health is being challenged in an unprecedented way by the 2019 coronavirus disease (COVID-19) pandemic. The systemic inflammatory response to the severe acute respiratory syndrome coronavirus-2 (SARS-CoV-2) infection is a hallmark of COVID-19, and most COVID-19 patients have abnormal inflammatory biomarkers^1^. C-reactive protein (CRP), an acute phase protein first described by Tillet and Francis, is synthesized by the liver in response to interleukin 6 (IL-6) and is a widely used biomarker of inflammation^2^. Elevated CRP concentrations are associated with cardiovascular disease and acute kidney injury^3^, and with inflammatory rheumatic diseases such as rheumatoid arthritis and gout^4^. CRP is also related to the severity of H1N1 influenza pneumonia patients^5^. Besides, most studies in recent years have pointed out that for COVID-19 patients, higher CRP concentrations are closely related to higher mortality *risk*^6–12^. However, some studies have pointed out that CRP may play a protective role in alveolitis, and in patients with acute lung injury, an increase in CRP levels is associated with a decrease in mortality^13^. Therefore, it is still controversial whether the increase of CRP in the early stage of COVID-19 can be used as a prognostic indicator. Moreover, most studies had small sample sizes and did not consider the curvilinear relationship between CRP and mortality. The aim of this retrospective cohort study was to determine whether elevated levels of CRP measured in the early stages of COVID-19 were associated with higher mortality in COVID-19 patients.

## Methods

This is a retrospective cohort study of all patients admitted to 4 hospitals within the Montefiore Health System between March 1 and April 16, 2020, with SARS-CoV-2 infection. Information on demographics, comorbidities, admission laboratory values, discharge, and mortality was identifified through a health care surveillance software package (Clinical Looking Glass; Streamline Health, Atlanta, GA) and review of the primary medical records. The design details in this study have been previously reported^14^.

### Data source

We downloaded the raw data free of charge from the DATADRYAD database provided by Eskandar et al. Source: Altschul, David (2021), Neurologic complications of COVID-19, Dryad, Dataset, <https://doi.org/10.5061/dryad.7d7wm37sz>. Under Dryad's Terms of Service, researchers can use this data for secondary analysis without infringing on the authors' rights.

### Study population

**Figure 1:**
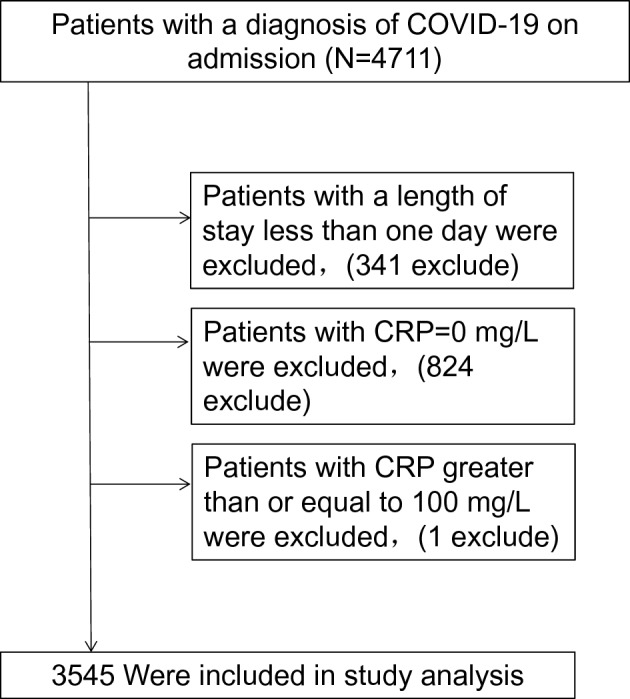
Flow chart of study population.

All patients with real-time reverse transcriptase PCR positive assay testing for SARS-CoV-2 RNA were included.For patients with multiple admissions, only the last reported was considered for analysis.Data were collected on May 7, 2020.Exclusion criteria were as follows: (1) patients who were not admitted or died before admission were excluded because they rarely had a complete laboratory study panel and a complete neurological evaluation could not be assessed, (2) CRP = 0 mg/L or Patients with CRP ≥ 100 mg/L. The flow chart of the study is shown in Fig. 1.

### Variables

The variable for this study was C-reactive protein, and the covariate was chosen based on our clinical experience, original studies, and other studies investigating risk factors for progression in COVID-19 patients. Based on the above principles, the following variables are therefore used as covariates: (1) Continuous variables: age, D-Dimer, temperature, oxygen saturation, mean arterial pressure, platelets, INR, BUN, creatinine, sodium, glucose, AST, WBC, ALT, lymphocytes, interleukin-6, ferritin, procalcitonin, troponin; (2) Categorical variables: ethnicity, myocardial infarction, peripheral vascular disease, congestive heart failure, cerebrovascular disease, dementia, chronic obstructive pulmonary disease, diabetes mellitus simple, renal disease, stroke.

### Outcomes

Te outcome of the study was all-cause mortality.

### Statistical analysis

Continuous variables were described as the means ± SD or median and interquartile ranges (IQR). Categorical data were presented as numbers and percentages. The diference according to the tertiles of the CRP was compared using one-way analysis of variance (ANOVA) for continuous data and chi-squared tests for categorical variables.

Calculations for Kaplan Meier survival were performed using Kaplan Meier analysis, and a Kaplan Meier curve was developed as a result.

**Figure 2:**
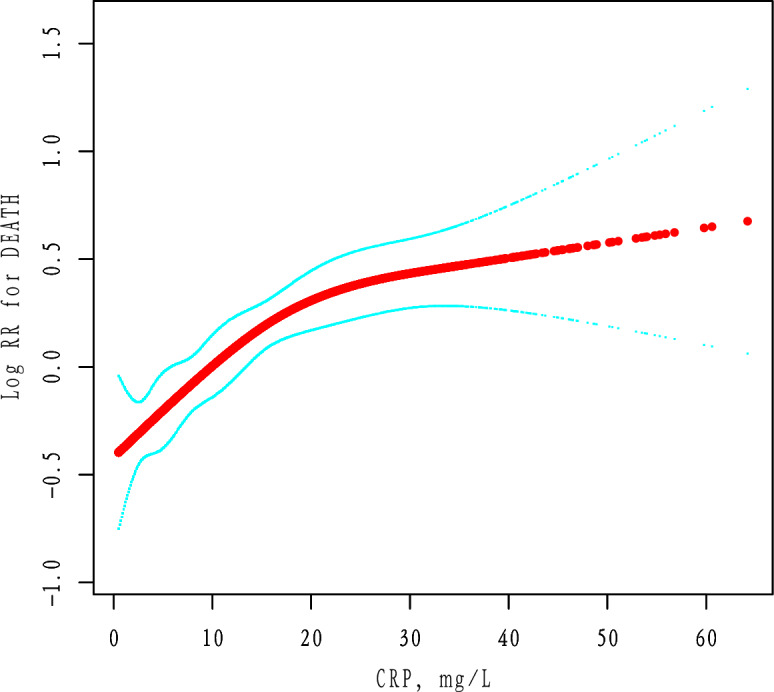
Associations between the CRP and mortality in all patients with Covid-19. A threshold, nonlinear association between the CRP and mortality was found in a generalized additive model (GAM). Red rad line represents the smooth curve fit between variables. Blue bands represent the 95% of confdence interval from the fit. Adjusted for age, D-Dimer, temperature, oxygen saturation, mean arterial pressure, platelets, INR, BUN, creatinine, sodium, glucose, AST, WBC, ALT, lymphocytes, interleukin-6, ferritin, procalcitonin, troponin, ethnicity, myocardial infarction, peripheral vascular disease, congestive heart failure, cerebrovascular disease, dementia, chronic obstructive pulmonary disease, diabetes mellitus simple, renal disease, stroke.

We used a generalized additive model (GAM) to investigate the dose–response relationship between the CRP and mortality (Fig. 2). Adjusted for age, D-Dimer, temperature, oxygen saturation, mean arterial pressure, platelets, INR, BUN, creatinine, sodium, glucose, AST, WBC, ALT, lymphocytes, interleukin-6, ferritin, procalcitonin, troponin, ethnicity, myocardial infarction, peripheral vascular disease, congestive heart failure, cerebrovascular disease, dementia, chronic obstructive pulmonary disease, diabetes mellitus simple, renal disease, stroke. Estimating the hazard ratio (HR) of death was done using Cox proportional hazards modeling .

We then used a two-piece-wise linear regression model to examine the threshold effect of the CRP on mortality (Table 3). The turning point for the CRP was determined using “exploratory” analyses, which is to move the trial turning point along the predefned interval and pick up the one which gave maximum model likelihood. We also performed a log-likelihood ratio test and compared the one-line linear regression model with the two-piece-wise linear model. All the statistical analyses were performed using the EmpowerStats (www.empowerstats.com, X&Y solutions, Inc. Boston MA) and R software version 3.6.1 (http://www.r-project.org)^15^.

### Ethics approval and consent to participate

The study received approval from Albert Einstein College of Medicine, Montefiore Medical Center ethical standards committee on human experimentation. Written informed consent was waived by the Albert Einstein College of Medicine, Montefiore Medical Center ethical standards committee given the retrospective design of the study. In addition to this, Methods of the study were performed in accordance with the relevant guidelines and regulations.

## Results

### Baseline characteristics

Data from 3545 patients were analyzed. The median age is 63.7 years (IQR54-76 years). Table 1 compares patient’s demographics, vital signs, laboratory results, and complications by tertile of CRP. Subjects in the highest tertile of CRP were older and had lower Oxygensaturation on admission than those in the lowest tertile of CRP (Table 1).

**Table 1:** Baseline characteristics and mortality according to the tertiles of the CRP (n = 3545).

Parameters	CRP, mg/L			P value
	Tertile 1 0.50–5.50 n = 1181	Tertile 2 5.60–14.90 n = 1176	Tertile 3 15.00–68.40 n = 1188	
Continuous				
Age, y	60.99 ± 17.18	64.52 ± 15.89	65.59 ± 15.14	< 0.001
D-Dimer, mg/L	1.00 (0.47–2.33)	1.40 (0.73–3.23)	2.51 (1.19–6.80)	< 0.001
Temperature, °C	37.14 ± 0.90	37.40 ± 0.90	37.49 ± 0.95	< 0.001
Oxygen saturation, %	95.43 ± 4.94	92.61 ± 7.00	89.56 ± 10.09	< 0.001
LOS, month	6.64 ± 6.25	8.45 ± 7.27	9.14 ± 7.67	< 0.001
Mean arterial pressure, mm Hg	84.87 ± 22.71	83.77 ± 20.92	78.50 ± 24.08	< 0.001
Platelets, k/mm^3^	232.19 ± 103.52	224.23 ± 89.94	236.00 ± 95.53	0.011
INR	1.06 ± 0.37	1.09 ± 0.33	1.16 ± 0.35	< 0.001
BUN, mg/dL	15.00 (10.00–28.00)	17.00 (10.00–32.00)	19.00 (11.00–39.00)	< 0.001
Creatinine, μmol/L	1.00 (0.77–1.53)	1.10 (0.81–1.78)	1.26 (0.90–2.25)	< 0.001
Sodium, mmol/L	138.27 ± 7.25	137.83 ± 7.87	137.98 ± 7.81	0.380
Glucose, mmol/L	113.00 (0.00–153.25)	118.00 (0.00–162.00)	121.00 (0.00–173.00)	0.281
AST, U/L	31.00 (22.00–48.00)	41.00 (28.00–63.00)	51.00 (34.00–77.00)	< 0.001
WBC count per mm^3^	7.30 ± 3.49	7.84 ± 3.63	9.77 ± 4.20	< 0.001
ALT, U/L	23.00 (15.00–37.00)	27.00 (18.00–42.75)	30.00 (19.00–47.00)	< 0.001
Lymphocytes per mm^3^	1.20 (0.80–1.60)	0.90 (0.70–1.30)	0.90 (0.60–1.20)	< 0.001
Interleukin-6, pg/mL	0.00 (0.00–15.90)	6.40 (0.00–40.70)	13.70 (0.00–75.10)	< 0.001
Ferritin, μg/L	308.50 (66.75–759.50)	652.00 (229.50–1416.50)	946.00 (440.00–1930.25)	< 0.001
Procalcitonin, ng/mL	0.10 (0.00–0.10)	0.10 (0.00–0.30)	0.30 (0.10–1.40)	< 0.001
Troponin, ng/mL	0.01 (0.01–0.01)	0.01 (0.01–0.02)	0.01 (0.01–0.04)	< 0.001
Categorical				
Black	423 (35.82%)	462 (39.29%)	418 (35.19%)	0.084
White	125 (10.58%)	112 (9.52%)	106 (8.92%)	0.383
Asian	30 (2.54%)	25 (2.13%)	36 (3.03%)	0.379
Latino	445 (37.68%)	423 (35.97%)	467 (39.31%)	0.245
Myocardial infarction	34 (2.88%)	58 (4.93%)	59 (4.97%)	0.016
Peripheral vascular disease	198 (16.77%)	172 (14.63%)	149 (12.54%)	0.005
Congestive heart failure	108 (9.14%)	155 (13.18%)	132 (11.11%)	0.008
Cerebrovascular disease	107 (9.06%)	143 (12.16%)	120 (10.10%)	0.043
Dementia	81 (6.86%)	98 (8.33%)	84 (7.07%)	0.336
Chronic obstructive pulmonary disease	65 (5.50%)	74 (6.29%)	76 (6.40%)	0.610
Diabetes mellitus simple	158 (13.38%)	188 (15.99%)	170 (14.31%)	0.191
Renal disease	192 (16.26%)	236 (20.07%)	209 (17.59%)	0.050
Stroke	16 (1.35%)	11 (0.94%)	18 (1.52%)	0.430
Mortality				
No	1040 (88.06%)	890 (75.68%)	697 (58.67%)	< 0.001
Yes	141 (11.94%)	286 (24.32%)	491 (41.33%)	

Data are expressed as the mean ± SD, median (interquartile range), or percentage.

### Mortality

The mortality rate was 918/3545 = 25.90% in our cohort. The mortality rate from the lowest tertile (0.50–5.50) to the highest (15.00–68.40) CRP was 141 (11.94%), 286 (24.32%), and 491 (41.33%) (Table 1).

### Unadjusted association between baseline variables and mortality

In the univariate COX regression analysis model, CRP, age, D-Dimer, oxygen saturation, mean arterial pressure, BUN, creatinine, sodium, glucose, AST, WBC, lymphocytes, interleukin-6 and Procalcitonin are all correlated with mortality, and have statistical significance, as shown in Table 2.

**Table 2:** The unadjusted association between baseline variables and mortality (n = 3545).

Exposure	Statistics	HR (95% CI)	P value
CRP, per 10 mg/L	12.63 (4.20–18.20)	1.31 (1.25, 1.38)	< 0.0001
CRP, per 10 mg/L Tertile			
Low	1181 (33.31%)	Reference	
Middle	1176 (33.17%)	1.53 (1.25, 1.87)	< 0.0001
High	1188 (33.51%)	2.36 (1.96, 2.85)	< 0.0001
Black			
No	2242 (63.24%)	Reference	
Yes	1303 (36.76%)	0.90 (0.79, 1.03)	0.1399
White			
No	3202 (90.32%)	Reference	
Yes	343 (9.68%)	1.14 (0.93, 1.40)	0.2033
Asian			
No	3454 (97.43%)	Reference	
Yes	91 (2.57%)	1.06 (0.74, 1.53)	0.7410
Latino			
No	2210 (62.34%)	Reference	
Yes	1335 (37.66%)	1.07 (0.94, 1.22)	0.3115
Myocardial infarction			
No	3394 (95.74%)	Reference	
Yes	151 (4.26%)	0.92 (0.67, 1.27)	0.6312
Peripheral vascular disease			
No	3012 (84.96%)	Reference	
Yes	519 (14.64%)	0.96 (0.79, 1.18)	0.7186
Congestive heart failure			
No	3150 (88.86%)	Reference	
Yes	395 (11.14%)	1.05 (0.86, 1.27)	0.6506
Cerebrovascular disease			
No	3175 (89.56%)	Reference	
Yes	370 (10.44%)	1.09 (0.89, 1.34)	0.4118
Dementia			
No	3282 (92.58%)	Reference	
Yes	263 (7.42%)	1.06 (0.84, 1.33)	0.6325
Chronic obstructive pulmonary disease			
No	3330 (93.94%)	Reference	
Yes	215 (6.06%)	1.07 (0.82, 1.39)	0.6177
Diabetes mellitus simple			
No	3029 (85.44%)	Reference	
Yes	516 (14.56%)	0.97 (0.81, 1.17)	0.7614
Renal disease			
No	2908 (82.03%)	Reference	
Yes	637 (17.97%)	1.15 (0.98, 1.35)	0.0917
Stroke			
No	3500 (98.73%)	Reference	
Yes	45 (1.27%)	1.25 (0.81, 1.92)	0.3201
Age, y	63.70 ± 16.21	1.04 (1.04, 1.05)	< 0.0001
Age, y Tertile			
Low	1127 (31.79%)	Reference	
Middle	1203 (33.94%)	2.17 (1.75, 2.68)	< 0.0001
High	1215 (34.27%)	4.03 (3.30, 4.94)	< 0.0001
D-Dimer, mg/L	1.55 (0.71–3.60)	1.04 (1.03, 1.05)	< 0.0001
D-Dimer, mg/L Tertile			
Low	1182 (33.34%)	Reference	
Middle	1177 (33.20%)	1.05 (0.87, 1.26)	0.5996
High	1186 (33.46%)	1.53 (1.29, 1.81)	< 0.0001
Temperature, °C	36.54 ± 5.67	1.01 (0.99, 1.02)	0.2363
Temperature, °C Tertile			
Low	1056 (30.42%)	Reference	
Middle	1249 (35.98%)	0.89 (0.76, 1.05)	0.1593
High	1166 (33.59%)	0.89 (0.76, 1.05)	0.1696
Oxygen saturation, %	90.70 ± 15.05	1.00 (0.99, 1.00)	0.0427
Oxygen saturation, % Tertile			
Low	1011 (29.09%)	Reference	
Middle	1007 (28.97%)	0.66 (0.55, 0.78)	< 0.0001
High	1458 (41.94%)	0.69 (0.60, 0.81)	< 0.0001
MAP, mm Hg	82.37 ± 22.78	0.99 (0.98, 0.99)	< 0.0001
Mean arterial pressure, mm Hg Tertile			
Low	1163 (32.81%)	Reference	
Middle	1183 (33.37%)	0.36 (0.31, 0.43)	< 0.0001
High	1199 (33.82%)	0.40 (0.34, 0.47)	< 0.0001
Platelets, k/mm^3^	235.58 ± 108.19	1.00 (1.00, 1.00)	0.1478
Platelets, k/mm^3^ Tertile			
Low	1159 (33.02%)	Reference	
Middle	1167 (33.25%)	0.97 (0.83, 1.13)	0.6909
High	1184 (33.73%)	0.86 (0.73, 1.01)	0.0643
INR Tertile			
Low	338 (9.63%)	Reference	
Middle	1080 (30.77%)	0.80 (0.61, 1.05)	0.1094
High	2092 (59.60%)	1.25 (0.98, 1.60)	0.0712
BUN, mg/dL	17 (10–34)	1.01 (1.01, 1.01)	< 0.0001
BUN, mg/dL Tertile			
Low	1099 (31.30%)	Reference	
Middle	1205 (34.32%)	1.16 (0.97, 1.40)	0.1061
High	1207 (34.38%)	2.03 (1.73, 2.38)	< 0.0001
Creatinine, μmol/L	1.10 (0.80–1.90)	1.04 (1.02, 1.06)	< 0.0001
Creatinine, μmol/L Tertile			
Low	1075 (30.63%)	Reference	
Middle	1260 (35.90%)	1.43 (1.17, 1.74)	0.0004
High	1175 (33.48%)	2.24 (1.87, 2.69)	< 0.0001
Sodium, mmol/L	135.69 ± 19.35	1.01 (1.00, 1.01)	
Sodium, mmol/L Tertile			
Low	999 (28.67%)	Reference	
Middle	1135 (32.57%)	0.86 (0.72, 1.03)	0.1058
High	1351 (38.77%)	1.31 (1.12, 1.53)	0.0007
Glucose, mmol/L	117 (0–165)	1.00 (1.00, 1.00)	0.0008
Glucose, mmol/L Tertile			
Low	1160 (33.05%)	Reference	
Middle	1167 (33.25%)	0.96 (0.81, 1.14)	0.6435
High	1183 (33.70%)	1.46 (1.25, 1.69)	< 0.0001
AST, U/L	40(27–65)	1.00 (1.00, 1.00)	< 0.0001
AST, U/L Tertile			
Low	1150 (32.76%)	Reference	
Middle	1163 (33.13%)	1.02 (0.86, 1.22)	0.7920
High	1197 (34.10%)	1.33 (1.13, 1.56)	0.0007
WBC count per mm^3^	8.75 ± 7.62	1.01 (1.01, 1.02)	< 0.0001
WBC count per mm^3^ Tertile			
Low	1158 (32.99%)	Reference	
Middle	1172 (33.39%)	1.19 (1.00, 1.40)	0.0448
High	1180 (33.62%)	1.31 (1.11, 1.53)	0.0012
ALT, U/L	27 (17–45)	1.00 (1.00, 1.00)	0.1157
ALT, U/L Tertile			
Low	1106 (32.16%)	Reference	
Middle	1162 (33.79%)	1.04 (0.88, 1.22)	0.6660
High	1171 (34.05%)	1.01 (0.85, 1.18)	0.9455
Lymphocytes per mm^3^	1.00 (0.70–1.40)	1.01 (1.00, 1.02)	0.0621
Lymphocytes per mm^3^ Tertile			
Low	1059 (30.17%)	Reference	
Middle	1096 (31.23%)	0.81 (0.69, 0.95)	0.0101
High	1355 (38.60%)	0.77 (0.66, 0.90)	0.0011
Interleukin-6, pg/mL	5 (0–41.54)	1.00 (1.00, 1.00)	0.0086
Interleukin-6, pg/mL Tertile			
Middle	2316 (66.67%)	Reference	
High	1158 (33.33%)	1.15 (1.01, 1.32)	0.0356
Ferritin, μg/L	628 (180–1402)	1.00 (1.00, 1.00)	0.0054
Ferritin, μg/L Tertile			
Low	1168 (33.28%)	Reference	
Middle	1172 (33.39%)	0.89 (0.75, 1.06)	0.1891
High	1170 (33.33%)	1.02 (0.87, 1.20)	0.8190
Procalcitonin, ng/mL	0.1 (0–0.5)	1.01 (1.01, 1.02)	< 0.0001
Procalcitonin, ng/mL Tertile			
Low	974 (28.32%)	Reference	
Middle	1315 (38.24%)	0.59 (0.48, 0.71)	< 0.0001
High	1150 (33.44%)	1.06 (0.90, 1.25)	0.4828
Troponin, ng/mL	0.01 (0.01–0.02)	1.27 (1.15, 1.40)	< 0.0001
Troponin, ng/mL Tertile			
Low	353 (10.06%)	Reference	
High	3157 (89.94%)	1.04 (0.81, 1.33)	0.7520

Data are expressed as the mean ± SD, median (interquartile range), or percentage.

**Figure 3:**
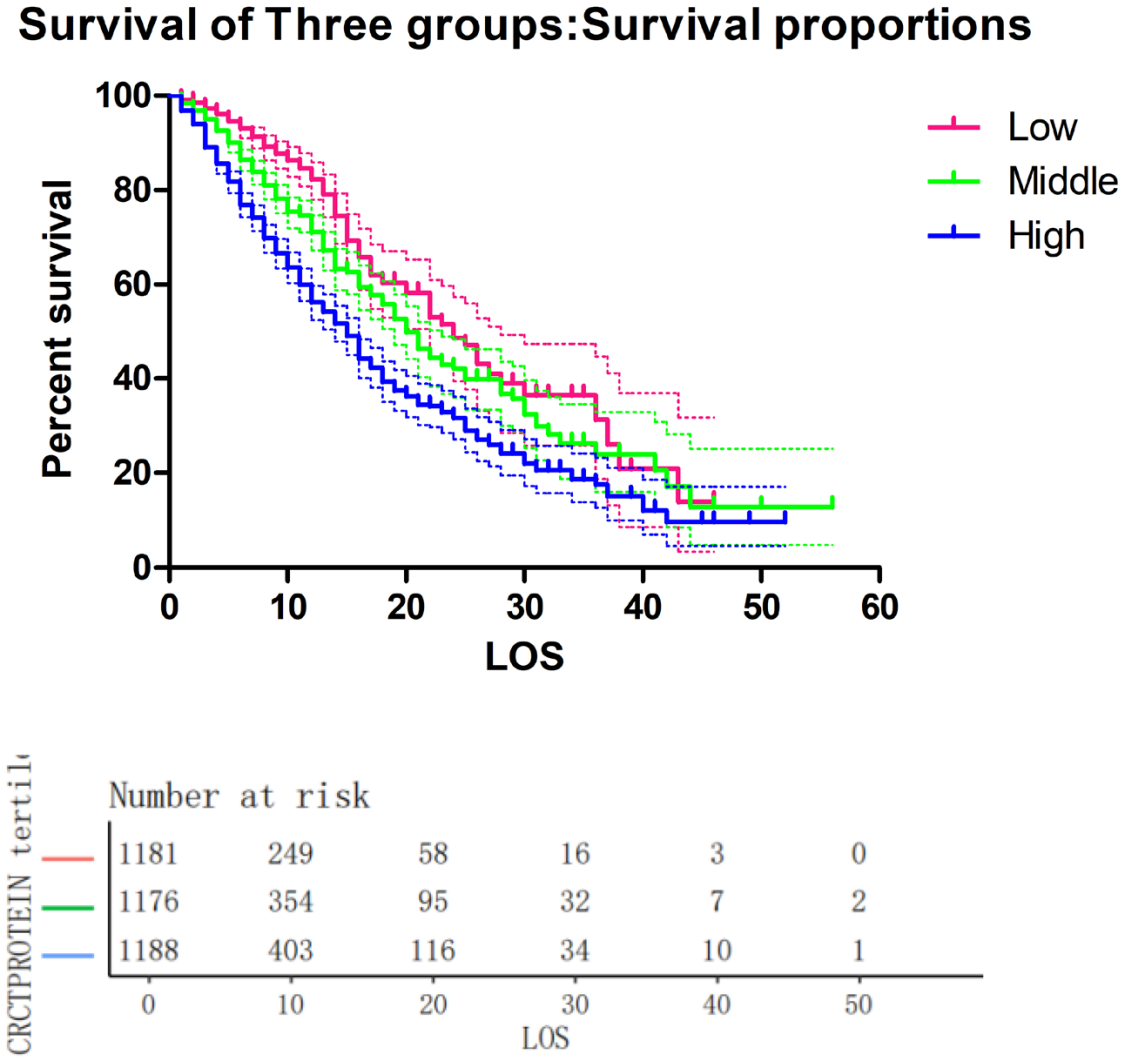
Kaplan–Meier survival curves for all-cause mortality stratified by CRP tertile.

In addition, we can intuitively see from that the higher the CRP, the lower the survival rate. See Fig. 3 for details.

### Identifcation of nonlinear relationship

We observed a nonlinear dose–response relationship between the CRP and mortality (Fig. 2 and Table 3). When the CRP was < 15.6 mg/L, the mortality rate increased with an adjusted HR of 1.57 (95% CI 1.30–1.91, *P* < 0.0001) for every 10 mg/L increment in the CRP. When the CRP was ≥ 15.6 mg/L, the mortality rate increased with an adjusted HR of 1.11 (95% CI 0.99–1.24, P = 0.0819) for every 10 mg/L increment in the CRP (Table 3).

**Table 3:** Treshold efect analysis of the CRP and mortality.

Models	Per-10 unit increase	
	HR (95% CI)	P value
Model I		
One line effect	1.23 (1.14, 1.33)	< 0.0001
Model II		
Turning point (K)	1.56	
CPR < K	1.57 (1.30, 1.91)	< 0.0001
CPR ≥ K	1.11 (0.99, 1.24)	0.0819
P value for LRT test*		0.007

Data were presented as HR (95% CI) P value; Model I, linear analysis; Model II, non-linear analysis. Adjusted for age, D-Dimer, temperature, oxygen saturation, mean arterial pressure, platelets, INR, BUN, creatinine, sodium, glucose, AST, WBC, ALT, lymphocytes, interleukin-6, ferritin, procalcitonin, troponin, ethnicity, myocardial infarction, peripheral vascular disease, congestive heart failure, cerebrovascular disease, dementia, chronic obstructive pulmonary disease, diabetes mellitus simple, renal disease, stroke. CI confdence interval, HR hazard ratio, LRT logarithm likelihood ratio test. *P < 0.05 indicates that model II is signifcantly diferent from Model I.

Using the generalized additive model, an inflection point between CRP and mortality was detected (Table 3). The linear regression model and a two-piece-wise linear regression model were compared, and the P value of the log-likelihood ratio test was 0.007. This result indicates that the two-piece-wise linear regression model should be used to fit the model.

## Discussion

This retrospective cohort study found a curved relationship and saturation effect between CRP and the mortality risk in 3545 patients infected with SARS-CoV-2 admitted to four hospitals within the Montefiore health system between March 1 and April 16, 2020. When the CRP was small, the mortality rate increased significantly with the increase of CRP. When CRP > 15.6 mg/L, with the increase of CRP, the mortality rate increases relatively flat. Few studies have shown a curvy relationship between CRP and mortality in patients with COVID-19.

C-reactive protein is recognized as a marker of systemic inflammation and severe infection. As an acute phase reactant, CRP binds to phosphorylcholine in pathogen and host cell membranes and acts as an opsonin to enhance phagocytosis and facilitate clearance. Ligand-bound C-reactive protein also efficiently activates the classical pathway of the complement system, an essential component of natural host defense^16^. Prior to the COVID-19 global pandemic, up to 90% of significantly elevated C-reactive protein concentrations were attributable to infectious pathogens, most commonly bacterial pathogens^17^. Elevated C-reactive protein concentrations have also been reported in severe viral infections, including H1N1 influenza pneumonia and now SARS-CoV-2 infection^5,18^.In a previous study of 95 COVID-19 patients, CRP For every 10 mg/L increase, the patient's risk of death increased by 11%, and C-reactive protein concentration was associated with mortality^9^. Smilowitz et al. reported that patients with COVID-19 who had high levels of C-reactive protein on admission were associated with higher mortality, and that patients with both high D-dimer and CRP had a greater risk of death^8^. Similarly, among 281 hypertensive COVID-19 patients hospitalized with COVID-19, those who died had higher levels of C-reactive protein compared with survivors^19^. Although the studies collected patients over different time periods, the trends were similar. In addition, the high mortality rate of COVID-19 is mainly related to the poor control of the inflammatory cascade and the subsequent acute respiratory distress syndrome (ARDS)^20^. However, some studies have pointed out that elevated plasma CRP levels in patients with acute respiratory distress syndrome are associated with reduced mortality, which seems to be contrary to the established views of previous scholars^13^. Therefore, the correlation between CRP and the prognosis of COVID-19 infection is controversial, and most of the previous similar studies had small sample sizes, and rarely analyzed the curve fitting relationship between CRP and the mortality rate of COVID-19 patients. This is exactly the problem that this study strives to solve.

C-reactive protein concentration reflects the degree of acute inflammatory response. The role of systemic inflammation in the pathogenesis of COVID-19 is still not fully understood. A causal relationship between the inflammatory response measured by CRP and poor prognosis remains speculative. However, the deleterious inflammatory response observed in some patients with COVID-19 is similar to macrophage activation syndrome and may independently lead to multiple organ damage in COVID-19^21,22^. Studies have shown that the application of glucocorticoids Decreases C-reactive protein concentrations in patients with ARDS^23^. A recent randomized trial of immunosuppression noted that hormone therapy reduced mortality in critically ill COVID-19 patients. The clinical benefit of immunosuppression further supports the hypothesis that inflammation from viral infection contributes to adverse outcomes in COVID-19^24^. In addition, thrombotic inflammation has been implicated as a mediator of adverse events in COVID-19. In response to inflammatory stress, thrombus inflammation dysregulates the normal antithrombotic function of the endothelium, leading to leukocyte recruitment, complement and platelet activation, and enhanced microvascular coagulation^25,26^. This is supported by autopsy studies of COVID-19 patients. In these studies, platelets with fibrin microthrombi were found in the microcirculation of the lung, kidney, liver, and heart^27^. Among them, pulmonary embolism has a high mortality rate and is very common in new coronary pneumonia. Therefore, the risk of poor prognosis associated with SARS-CoV-2 infection increases when patients experience concomitant elevated CRP concentrations. However, what we did not expect is that there is a saturation effect between CRP and mortality. So far, the author has not found relevant literature to explain this phenomenon.

Among COVID-19 patients, higher C-reactive protein levels were associated with higher mortality risk with a saturation effect. This finding has interesting potential clinical and research implications. To the best of our knowledge, there are few clear references to the curvilinear relationship between CRP and mortality risk in the early stages of COVID-19. Compared with the linear regression relationship, the curve relationship in this study can more scientifically and accurately reflect the judgment value of CRP level on the mortality risk of patients with COVID-19.

### Study limitations

A common problem in observational studies is unmeasured confounders. As shown in Table 1, subjects in the highest tertile of CRP were older and had lower Oxygensaturation compared with subjects in the lowest tertile of CRP. These differences may be indicative of unmeasured confounders, such as income and health insurance, which may affect the mortality risk. Although race, length of hospital stay, and age were included in the data set, the effect of unmeasured confounders on HR could not be estimated. In addition, the first measured CRP concentration on admission was used for the primary analysis. The admission physician's knowledge of the initial CRP concentration may have altered subsequent patient care and may have confounded the results. Although there are no data on steroid use, few patients may have received steroids prior to the initial CRP measurement during hospitalization. In some cases, steroids or the IL-6 inhibitor tocilizumab are used in critically ill patients. However, the timing of administration, duration of use, and dosage of these drugs were not recorded in this dataset.

In addition, the lack of information on interventions during the initial stabilization period may have influenced the relationship between CRP levels and survival. It is noteworthy that the potential of the intervention would be biased towards reducing mortality, leading to an underestimation of the association between CRP levels and mortality, but this reinforces the positive findings of this study.

## Conclusions

The study identified a nonlinear dose–response relationship between CRP and mortality. For patients with COVID‐19, the association between the CRP and the mortality risk was curve and had a saturation effect. When the CRP was small, the mortality rate increased significantly with the increase of CRP. When CRP > 15.6 mg/L, with the increase of CRP, the mortality rate increases relatively flat.

### Supplementary Information

Supplementary Information 1.

Supplementary Information 2.

Supplementary Information 3.

Supplementary Information 4.

## Data Availability

The datasets generated and/or analysed during the current study are available in the DATADRYAD repository, https://doi.org/10.5061/dryad.7d7wm37sz.
