# Supplementary material for: Association of C-reactive protein with mortality in Covid-19 patients: a secondary analysis of a cohort study
Source: Sci Rep. 2023 Nov 21;13:20361. doi: 10.1038/s41598-023-47680-x (PMC10663442; doi:10.1038/s41598-023-47680-x)
Supplement: Supplementary file 1 — Supplementary Information 1. [file 41598_2023_47680_MOESM1_ESM.doc]

**Supplementary 1.** Association between CRP and mortality in different model

| **Variable** | **Non-adjusted (HR, 95% CI, P)** | **Adjust I (HR, 95% CI, P)** | **Adjust II (HR, 95% CI, P)** |
| --- | --- | --- | --- |
| CRP, per 10 mg/L | 1.31 (1.25, 1.38) <0.0001 | 1.22 (1.16, 1.29) <0.0001 | 1.23 (1.14, 1.33) <0.0001 |
| CRP, per 10 mg/L tertile |  |  |  |
| Low | Reference | Reference | Reference |
| Middle | 1.53 (1.25, 1.87) <0.0001 | 1.34 (1.09, 1.65) 0.0055 | 1.28 (1.01, 1.61) 0.0409 |
| High | 2.36 (1.96, 2.85) <0.0001 | 1.92 (1.58, 2.34) <0.0001 | 1.91 (1.51, 2.43) <0.0001 |
| P for trend | <0.0001 | <0.0001 | <0.0001 |

**Notes:** Non-adjusted model: we did not adjust other covariates. Model I: we adjust age, Mean arterial pressure, temperature, oxygen saturation. Model II: we adjust age, D-Dimer, temperature, oxygen saturation, mean arterial pressure, platelets, INR, BUN, creatinine, sodium, glucose, AST, WBC, ALT, lymphocytes, interleukin-6, ferritin, procalcitonin, troponin, ethnicity, myocardial infarction, peripheral vascular disease, congestive heart failure, cerebrovascular disease, dementia, chronic obstructive pulmonary disease, diabetes mellitus simple, renal disease, stroke.

**Abbreviations:** CI, confidence.
