# Supplementary material for: Association of C-reactive protein with mortality in Covid-19 patients: a secondary analysis of a cohort study
Source: Sci Rep. 2023 Nov 21;13:20361. doi: 10.1038/s41598-023-47680-x (PMC10663442; doi:10.1038/s41598-023-47680-x)
Supplement: Supplementary file 2 — Supplementary Information 2. [file 41598_2023_47680_MOESM2_ESM.doc]

**Supplementary 2.** Subgroup analyses between CRP and mortality

| Sub-group | N | DEATH |
| --- | --- | --- |
| X= CRP |  |  |
| MI |  |  |
| No | 3394 | 1.31 (1.25, 1.38) <0.0001 |
| Yes | 151 | 1.30 (0.99, 1.69) 0.0558 |
| CHF |  |  |
| No | 3150 | 1.30 (1.23, 1.37) <0.0001 |
| Yes | 395 | 1.40 (1.23, 1.60) <0.0001 |
| CVD |  |  |
| No | 3175 | 1.30 (1.23, 1.37) <0.0001 |
| Yes | 370 | 1.47 (1.25, 1.72) <0.0001 |
| DEMENT |  |  |
| No | 3282 | 1.31 (1.24, 1.38) <0.0001 |
| Yes | 263 | 1.33 (1.14, 1.56) 0.0003 |
| COPD |  |  |
| No | 3330 | 1.31 (1.24, 1.38) <0.0001 |
| Yes | 215 | 1.29 (1.09, 1.54) 0.0035 |
| DM.SIMPLE |  |  |
| No | 3029 | 1.32 (1.25, 1.39) <0.0001 |
| Yes | 516 | 1.24 (1.07, 1.43) 0.0039 |
| RENAL.DISEASE |  |  |
| No | 2908 | 1.32 (1.25, 1.39) <0.0001 |
| Yes | 637 | 1.28 (1.14, 1.44) <0.0001 |
