# Supplementary material for: Association of C-reactive protein with mortality in Covid-19 patients: a secondary analysis of a cohort study
Source: Sci Rep. 2023 Nov 21;13:20361. doi: 10.1038/s41598-023-47680-x (PMC10663442; doi:10.1038/s41598-023-47680-x)
Supplement: Supplementary file 3 — Supplementary Information 3. [file 41598_2023_47680_MOESM3_ESM.doc]

**Supplementary 3.** Association between CRP and mortality in different models for raw data

| **Variable** | Non-adjusted **(HR, 95% CI, P)** | Adjust I **(HR, 95% CI, P)** | Adjust II **(HR, 95% CI, P)** |
| --- | --- | --- | --- |
| CRP, per 10 mg/L | 1.19 (1.14, 1.25) <0.0001 | 1.11 (1.05, 1.17) 0.0002 | 1.12 (1.06, 1.19) <0.0001 |

**Notes:** Non-adjusted model: we did not adjust other covariates. Model I: we adjust age, Mean arterial pressure, temperature, oxygen saturation. Model II: we adjust age, D-Dimer, temperature, oxygen saturation, mean arterial pressure, platelets, INR, BUN, creatinine, sodium, glucose, AST, WBC, ALT, lymphocytes, interleukin-6, ferritin, procalcitonin, troponin, ethnicity, myocardial infarction, peripheral vascular disease, congestive heart failure, cerebrovascular disease, dementia, chronic obstructive pulmonary disease, diabetes mellitus simple, renal disease, stroke.

**Abbreviations:** CI, confidence.
