# Supplementary material for: Association of C-reactive protein with mortality in Covid-19 patients: a secondary analysis of a cohort study
Source: Sci Rep. 2023 Nov 21;13:20361. doi: 10.1038/s41598-023-47680-x (PMC10663442; doi:10.1038/s41598-023-47680-x)
Supplement: Supplementary file 4 — Supplementary Information 4. [file 41598_2023_47680_MOESM4_ESM.doc]

**Supplementary 4.** multivariate results including many factors and mortality

| **Parameters** | HR | 95% CI lower | 95% CI upper | P |
| --- | --- | --- | --- | --- |
| Myocardial infarction | 0.7238 | 0.5199 | 1.0078 | 0.0556 |
| Renal disease | 1.2126 | 1.0268 | 1.4320 | 0.0231 |
| Stroke | 2.8854 | 1.0150 | 8.2023 | 0.0468 |
| Age, y | 1.0382 | 1.0327 | 1.0438 | 0.0000 |
| Oxygen saturation, % | 0.9854 | 0.9794 | 0.9915 | 0.0000 |
| Temperature, °C | 1.0531 | 1.0326 | 1.0740 | 0.0000 |
| Mean arterial pressure, mm Hg | 0.9861 | 0.9836 | 0.9886 | 0.0000 |
| Platelets, k/mm^3^ | 0.9990 | 0.9983 | 0.9997 | 0.0047 |
| BUN, mg/dL | 1.0044 | 1.0021 | 1.0066 | 0.0001 |
| Sodium, mmol/L | 1.0036 | 1.0007 | 1.0065 | 0.0155 |
| Glucose, mmol/L | 1.0007 | 1.0002 | 1.0012 | 0.0045 |
| AST, U/L | 1.0002 | 1.0000 | 1.0003 | 0.0148 |
| WBC count per mm^3^ | 0.9887 | 0.9734 | 1.0041 | 0.1504 |
| Lymphocytes per mm^3^ | 1.0269 | 1.0073 | 1.0469 | 0.0070 |
| Troponin, ng/mL | 1.3227 | 1.1787 | 1.4844 | 0.0000 |
